# Construction Notes

**Published:** 1907-03-30

**Authors:** 


### CONSTRUCTION NOTES.

### LANCASTER INFIRMARY.

Some much-needed additions to the buildings as well as improvements have been made, including the Lady Ashton Memorial Room for Light Treatment, the gift of the tenantry, employees, and servants of Lord Ashton, in memory of the late Lady Ashton, which was opened with fitting ceremony on July 7, 1906; also a sterilising room, special surgical requirements, and external fire-escapes. The Lady Ashton Memorial Room has been fully equipped, thanks to the generosity of a few friends who provided either apparatus or money for the purpose.

### LEICESTER INFIRMARY.

The annual report shows that the past has been a year of remarkable progress in several departments. The most striking feature has been the remarkable headway made with the extensive scheme of enlargement and improvement. This has already cost over £45,000, and proved an incalculable boon. It is now being supplemented by the reconstruction of the south-east wing, and that, in its turn, is to be followed by the erection of a nursing home at a cost of £20,000, and the improvement of the laundry. The forward movement, thanks largely to the indomitable enterprise of Sir Edward Wood, is still in full flood, and bids fair to transform the infirmary into a model of its kind.

### VICTORIA HOSPITAL, KINGSTON.

The accommodation of the hospital, and consequently its power to minister to the sick and suffering, have been increased twofold by the generous action of Mr. W. E. Green, of Devonshire House, Strawberry Vale, who has extended the two main wards so as to accommodate six additional beds in each, thus increasing the number of beds from ten beds and two cots to twenty-two beds and two cots. In addition, Mr. Green has built a sitting-room and two additional bedrooms for the nursing staff, rearranged the hot-water supply, carried out other improvements in the kitchen department, and paved the yard.

### OXFORD EYE HOSPITAL.

During the year very considerable improvements have been completed in the ground floor accommodation of the hospital. A new surgery has been built and properly equipped. The committee room (to which a small lavatory was added) has been converted into a consulting room, thus securing privacy and proper facility for the examination of patients. It has been fully provided with optical and electrical apparatus. The old surgery has been made into the much-needed larger and more convenient dispensary; the old dispensary has become an office for the visitors and matron and for storing the records of patients' cases. In addition to these alterations an apparatus has been installed for warming the corridors and the rooms.

### SOUTH DEVON HOSPITAL.

Serious attention is being given to the enlargement of the pathological department and the provision of necessary accommodation for the application of Finsen light treatment within the hospital. The medical board has pressed upon the committee the great importance of these improvements, plans for which are under consideration. Gill ward, containing seven beds, has been opened during the year for male surgical cases; and, consequent upon the many applications for the admission of children, the committee has set aside John Hay side ward for the use of six additional medical children, and has opened Hains side ward for the accommodation of the female surgical patients originally treated in John Hay side ward. At the request of the surgeons the committee has remodelled and refitted the sterilising-room, which, by the removal of a partition, has been enlarged. A new Bruce-Clarke steriliser and an instrument steriliser have been fitted, the floor laid with the Eubœolith flooring, and ample cupboard accommodation supplied. The drains of the institution have also been reconstructed.

### KINGSWOOD HOSPITAL.

The Handel Cossham Hospital at Lodge Hill, Kingswood, is to be opened early in June. Workmen are busily engaged on the interior and exterior, and the main drive to the institution is rapidly assuming a park-like aspect. The site is a healthy one, it being some 300 yards above sea-level. The handsome clock tower, with its four illuminated dials, forms a landmark for many miles around, and the Cathedral chimes with which it is fitted are greatly appreciated. The hospital has a handsome appearance, being built in the Early Georgian style of architecture, with red pennant facing stone and Bath stone dressings. The main entrance has a smart triangular facing, supported by coupled Corinthian columns, and the donor's name is inscribed on the architecture. The tower is octagonal, and rises to the height of about 100 feet. The building ground occupies about four acres. The main wards are 50 feet by 25 feet, and have been planned for receiving ten beds in each. On the ground floor there is a fine operating theatre paved with mosaic, and attached are anæsthetic and sterilising rooms. There is splendid accommodation for the officials and surgeons, and also capacious corridors, giving access to the various departments; whilst at the massive entrance gates a lodge has been erected. The cost of the building will be £27,190, and there is about £80,000 to be utilised for the endowment.

### BIRMINGHAM CHILDREN'S HOSPITAL.

The chief topic of the speeches at the annual meeting of the Birmingham Children's Hospital was the urgent necessity which has arisen for the entire rebuilding of the present institution in Broad Street. The medical staff, in their report, said they felt it a matter of duty to urge the reconstruction of the hospital on a modern and larger plan. The death-rate among the in-patients was 17.02. The increasing death-rate of the past five years had been the subject of inquiry by a sub-committee, which found that it coincided with a proportionate increase in the number of admissions of children under the age of two years, in whom the death-rate from acute illness was very high. Dr. Robertson, the medical officer of health for the city, on the invitation of the Committee of Management, had inspected the hospital and drawn up a report, which, while not actually condemnatory, yet pointed out the structural and hygienic defects of the buildings at Broad Street, which disposed to repeated outbreaks and the spread of infectious diseases amongst the patients and nursing staff, in spite of every precaution. Dr. Robertson's views were in substantial agreement with those expressed in the annual report of the medical staff for 1905: "It is to be feared that until the hospital is reconstructed on a modern basis little can be done to avoid such outbreaks." A committee has, therefore, been appointed to consider the whole question, and it is hoped that within a short time it will be possible to submit for consideration an outline of the proposed scheme.

